# NIST Standard Reference Material 3600: Absolute Intensity Calibration Standard for Small-Angle X-ray Scattering

**DOI:** 10.1107/S1600576717001972

**Published:** 2017-03-07

**Authors:** Andrew J. Allen, Fan Zhang, R. Joseph Kline, William F. Guthrie, Jan Ilavsky

**Affiliations:** a Materials Measurement Science Division, National Institute of Standards and Technology, 100 Bureau Drive, Gaithersburg, MD 20899, USA; b Materials Science and Engineering Division, National Institute of Standards and Technology, 100 Bureau Drive, Gaithersburg, MD 20899, USA; c Statistical Engineering Division, National Institute of Standards and Technology, 100 Bureau Drive, Gaithersburg, MD 20899, USA; d X-ray Science Division, Advanced Photon Source, Argonne National Laboratory, 9700 South Cass Avenue, Argonne, IL 60439, USA

**Keywords:** small-angle X-ray scattering, absolute scattering intensity calibration, glassy carbon, standard reference materials

## Abstract

The certification of a new standard reference material for small-angle scattering [NIST Standard Reference Material (SRM) 3600: Absolute Intensity Calibration Standard for Small-Angle X-ray Scattering (SAXS)], based on glassy carbon, is presented. Creation of this SRM relies on the intrinsic primary calibration capabilities of the ultra-small-angle X-ray scattering technique. This article describes how the intensity calibration has been achieved and validated in the certified *Q* range, *Q* = 0.008–0.25 Å^−1^, together with the purpose, use and availability of the SRM. The intensity calibration afforded by this robust and stable SRM should be applicable universally to all SAXS instruments that employ a transmission measurement geometry, working with a wide range of X-ray energies or wavelengths. The validation of the SRM SAXS intensity calibration using small-angle neutron scattering (SANS) is discussed, together with the prospects for including SANS in a future renewal certification.

## Abstract 2

The certification of a new NIST standard reference material (SRM) for the calibration of small-angle X-ray scattering intensity is described, including the purpose, use and applicability of the SRM together with limitations and uncertainties in the intensity calibration provided.

## 1. Introduction and background   

Small-angle X-ray and neutron scattering (SAXS and SANS) methods are widely used to achieve a quantitative microstructure characterization that is statistically representative of a given sample material. SAXS or SANS data contain information regarding the sizes, shapes, concentrations and spatial arrangements of the inhomogeneities present (*e.g.* nanoparticles in a suspension, pores in a catalyst, precipitates in an alloy) and also regarding their specific surface areas (Guinier & Fournet, 1955 ▸; Kostorz, 1979 ▸; Glatter & Kratky, 1982 ▸). However, absolute intensity calibration of SAXS or SANS data, normalized both to the incident beam intensity and to sample volume, is a critical requirement for the quantitative determination of volume fraction (or porosity) and surface area information for nanoscale and mesoscale structures within advanced technological materials. Indeed, it is these details of the microstructure that frequently determine the key properties of a material and hence its performance in specific applications. Direct measurement of the scattering probability of a given sample material requires calibration of the weak scattered beam intensity relative to that of the incident beam. In practice, this often calls for more than eight decades in detector linear intensity dynamic range, which is beyond the instrumental capabilities of typical two-dimensional detectors used in most SAXS or SANS instruments. Usually, intensity calibration is carried out using one of three methods. (1) Intensity calibration is achieved with reference to a model fit (*e.g.* Guinier law or Zimm plot) to data collected over a restricted *Q* range from a ‘secondary’ standard (Wignall & Bates, 1987 ▸). (2) A ‘primary’ intensity calibration (Zemb *et al.*, 2003 ▸; Dreiss *et al.*, 2006 ▸) is accomplished using a series of previously calibrated attenuators to circumvent the detector intensity dynamic range issue. (3) Calibration is achieved through comparison with a ‘primary’ scattering standard such as water, where fundamental arguments, combined with physical measurements of compressibility at the measurement temperature, predetermine a scattering intensity that is approximately independent of scattering angle (Orthaber *et al.*, 2000 ▸). Disadvantages of these approaches are as follows: model results from a secondary standard will vary for experimental conditions different from those used to calibrate the standard; the absorption of calibrated attenuators can vary for different wavelengths, wavelength dispersion or instrument geometry; and water calibrations generally require very long measuring times (and the scattering intensity is not completely independent of the scattering angle in any case). In fact, most intensity calibration is ‘local’ to individual instruments or research institutions, with significant variations in calibrated results. For the increasing number of industrial-laboratory-based SAXS instruments that support new biomedical, pharmaceutical or nanotechnology development, there is frequently no intensity calibration at all.

Especially for laboratory-based SAXS instruments, there is clearly a need for a scattering intensity standard, calibrated and certified using a primary method (as defined below), where the measured scattering intensity (with a data collection time comparable to those for typical samples) can simply be compared with a certified calibration curve, without the need to fit a model in order to determine a calibration factor. This measured model-independent calibration factor can then be applied to all sample data measured using the same instrument configuration and under the same conditions, with the sample thickness and sample transmission (*i.e.* attenuation) as the only other independently measured parameters required (Zhang *et al.*, 2010 ▸). To develop an absolute intensity calibration standard reference material for SAXS, which meets the requirements set out above, two main conditions must be met: (i) a primary calibration measurement is required where the detector linear dynamic range is sufficient to register accurately both the incident beam intensity and the small-angle scattering intensity across the SAXS instrument range of measurement; and (ii) a stable scattering standard must be identified that provides significant scattering intensity over the range of SAXS instruments in general use.

**Figure 1:**
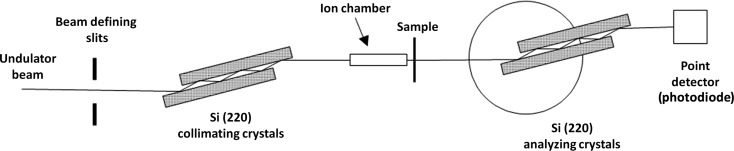
Schematic of APS USAXS measurement, Argonne National Laboratory.

In recent years, the development and application of Bonse–Hart crystal optics to small-angle scattering has enabled a primary intensity calibration measurement to be developed and its reliability established (Bonse & Hart, 1965 ▸; Long *et al.*, 1991 ▸). Fig. 1 ▸ shows a schematic of the synchrotron-based Bonse–Hart instrument at the Advanced Photon Source, Argonne National Laboratory, used in the present work (Ilavsky *et al.*, 2009 ▸, 2013 ▸). X-ray crystal optics are used both to define the collimated monochromatic incident beam (collimating crystals) and to determine the small-angle scattering intensity as a function of *Q* (analyzing crystals), where *Q* = (4π/λ)sinθ, λ is the X-ray wavelength and θ is half of the scattering angle, φ. This is done by rotating the analyzing crystal monolith through and away from the orientation where the Bragg condition at *Q* = 0 is satisfied. For a given rotation angle, φ, measured from that at *Q* = 0 where the Bragg condition for diffraction through the analyzing crystals is satisfied for the incident beam, only X-rays that have been scattered by the scattering angle φ now satisfy the Bragg condition. Because the intrinsic *Q* resolution is given by the Darwin width of the crystal optics, this configuration permits access to significantly lower *Q* values than most conventional SAXS instruments – hence ultra-small-angle X-ray scattering (USAXS). A key point is that the instrument incorporates an X-ray photodiode point detector (Jemian & Long, 1990 ▸) to collect the entire beam intensity diffracted by the analyzing crystals as a function of the scattering angle. Such a photodiode detector has a ten-decade intensity linear dynamic range, which is sufficient to capture both the weak SAXS intensity and the full intensity of the primary synchrotron X-ray beam within a single scan, without distortions arising from detector saturation or the need to use X-ray attenuators. Thus, although well suited to the low-*Q* regime, a USAXS instrument of this design can measure acceptable SAXS intensities to *Q* values well within the range of many conventional pinhole SAXS instruments. Meanwhile, an ion chamber placed before the sample records any temporal variations in the incident beam flux and is used to normalize out any corresponding temporal fluctuations in the photodiode signal not associated with the sample. These instrument attributes permit direct ‘primary’ calibration of the scattering intensity based on the fundamental definition of the differential scattering cross section per unit sample volume, dΣ/dΩ, defined as the probability per unit incident X-ray flux and per unit sample volume of scattering into unit solid angle about a direction associated with a given scattering vector, **Q**. As in all diffraction and elastic scattering, the direction of **Q** (with magnitude *Q*) bisects the incident and scattered beam directions, but for small-angle scattering it is approximately within the sample plane. The USAXS instrument used here has routinely provided such primary absolute intensity calibration reliably for many years. Since it does not require a scattering intensity calibration standard of its own, this instrument is well suited for certifying such a calibration standard for use elsewhere.

Previous work has established the feasibility of using glassy carbon as a stable SAXS intensity calibration standard, and various glassy (or vitreous) carbons have been recognized as potential intensity calibration standards for SAXS measurements (Dreiss *et al.*, 2006 ▸; Fan *et al.*, 2010 ▸; Zhang *et al.*, 2010 ▸). This is because the glassy carbon microstructure can be controlled depending on the starting polymer from which it is made, and it can produce significant small-angle scattering from its pore structure over a large part of the *Q* range of interest for SAXS or SANS (Craievich, 1976 ▸). Furthermore, glassy carbon samples exhibit minimal spatial variability in their microstructure and can be measured under ambient conditions. Glassy carbons are formed by the pyrolysis of a wide variety of polymers (Jenkins & Kawamura, 1971 ▸). Pyrolysis causes the polymer to transform directly into a carbon form (glassy carbon) that is both hard and brittle, unlike soft graphitic forms of carbon, with a final morphology and density dependent on the chemical composition and morphology of the starting polymer, together with the details of the pyrolytic heat treatment. In the case of a phenolic resin (for example), carbonization occurs through (i) intermolecular cross-link formation between hydroxyl groups within phenolic nuclei and methyl bridge formation between nuclei, together with elimination of water complexes up to ∼773 K; (ii) formation of randomly oriented and tangled aromatic ribbon molecules; and (iii) densification of the structure at higher temperatures with elimination of hydrogen and formation of intermolecular cross-links between the ribbons. The final steps result in the formation of a porous network of tangled aromatic ribbons cross-linked by highly strained C—C covalent bonds. The scattering contrast between the solid ribbons and the pore spaces provides the small-angle scattering intensity across the required *Q* range. A common attribute of hard glassy carbons is that negligible porosity is accessible to the exterior, so that they do not take up moisture or other sorbents from the environment, and a robust, stable standard can be developed, which can be used under ambient conditions (or under vacuum) without long-term degradation issues limiting its service life.

In the sections that follow, we describe how the glassy carbon calibration NIST Standard Reference Material (SRM) 3600 was selected and a calibration standard inventory established. We describe the development and certification of the absolute intensity calibration curve for SAXS dΣ/dΩ *versus Q* using USAXS measurements, together with evaluation of the associated uncertainties attributable to repeated measurement, sample variability and instrument setup. We also describe use of the new SRM in conventional SAXS measurements incorporating a two-dimensional area detector, and its validation using independent SANS measurements. Although the certified SAXS intensity calibration has been validated using SANS, this SRM is currently certified only for SAXS (including USAXS). This is because we are not able to verify at the present time that the intensity calibration will hold for all SANS instrument configurations in general use (May *et al.*, 2000 ▸).

## 2. Measurements to certify the SAXS absolute intensity calibration standard as a NIST SRM   

NIST SRMs are not usually the direct products of ‘round robin’ measurements made independently by multiple institutions, even though ‘round robin’ measurements did form part of the preliminary work that established the potential of glassy carbon as a SAXS intensity calibration standard (Zhang *et al.*, 2010 ▸). Rather, an inventory of SRM units is prepared, for which measurements of the desired property traceable to NIST primary standards have been made, complete with a full evaluation of the sources of uncertainty and their magnitudes (Taylor & Kuyatt, 1994 ▸).

### 2.1. Sample preparation   

Glassy carbon feedstock material was procured from Alfa Aesar, Ward Hill, MA.^1^ Alfa Aesar is a Johnson Matthey company (Johnson Matthey Co., London, UK). This was in the form of four Type 2 glassy carbon plates (Alfa Aesar product No. 38021), taken from the same lot, each plate with dimensions of 100 mm × 100 mm × 1 mm thick (nominal). The pyrolysis temperature used in the manufacture of this glassy carbon is 3273 K, which has been shown to be sufficient to remove virtually all hydrogen-containing polymer precursors (Cappelletti, 2016 ▸). The composition of the glassy carbon plates is rated to be 100% carbon by mass, and all four glassy carbon plates were from the same batch, as confirmed in a company-supplied Certificate of Analysis. Given that the theoretical (X-ray) density of carbon is 2.25 g cm^−3^ while the Certificate of Analysis indicates a bulk density for the glassy carbon plate of 1.42 g cm^−3^, this implies that the total internal porosity of the glassy carbon is 36.9%, distributed across the extended scale range of interest. The Certificate of Analysis confirms that no significant porosity is accessible from the surface. The exact thickness of each plate is in fact slightly greater than the nominal 1 mm specified, and closer to 1.05 mm.

Each of the four glassy carbon plates was sectioned into one hundred 10 × 10 mm (nominal) square coupons (400 coupons in all) as follows: (i) wax was applied to one side of each glassy carbon plate and then gentle heating was used to effectively ‘glue’ the plate to a flat metal surface on cooling; (ii) ten straight cuts were made across each plate in each of two orthogonal directions using a water-cooled diamond saw to create one hundred ∼10 mm-square pieces; (iii) the assembly was warmed to remove specimens from the metal surface and the wax was then cleaned off in acetone; (iv) for each plate, the one hundred ∼10 × 10 mm coupons were oven-heated for 12 h at 393 K (120°C) to remove any residue accumulated on the specimens during cutting; and finally (v) each coupon was packaged into an individual plastic membrane box container and labeled with a serial number unique to each SRM unit, each of which comprises a single glassy carbon coupon.

### 2.2. Repeatability and sample variation   

**Figure 2:**
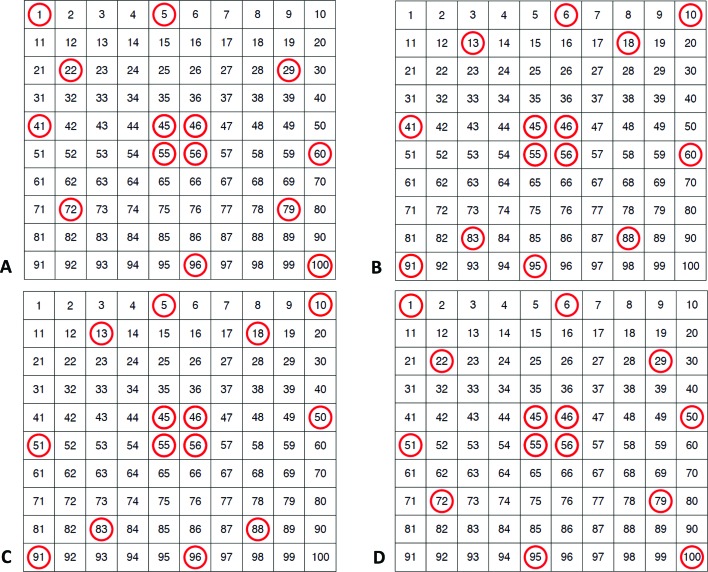
Maps showing locations of glassy carbon test coupons from plates A, B, C and D.

A selection of test coupons was made from the SRM inventory to check for any variations in calibrated SAXS intensity with the position within any one glassy carbon plate from which the coupons were cut, and for any variations for coupons cut from different plates, here designated A to D. Glassy carbon coupons were selected from each plate: four from central locations, four from the four edges, two from opposite corners, and four from positions intermediate between the center and edges of each plate (56 coupons in all). Omitting the corners, this specimen choice approximates a central composite experiment design and was designed to test for any systematic or random variations in the SAXS intensity with position or plate. Maps showing the test coupons selected are presented in Fig. 2 ▸.

The thickness of each test coupon was measured with a micrometer. The mean thickness measured with its standard deviation uncertainty was 1.055 ± 0.012 mm. The mean coupon thickness with 95% expanded uncertainty is 1.055 ± 0.025 mm on the basis of computations using a 95% confidence coverage factor of *k* = 2.004 obtained from the Student’s *t* distribution with 55 degrees of freedom^2^ (Taylor & Kuyatt, 1994 ▸; JCGM, 2008 ▸). We note that this represents a 95% confidence fractional uncertainty of ±2.28%. The thickness standard uncertainty propagates directly to the fractional standard uncertainty in the calibrated SAXS intensity for a thickness of 1.055 mm, which should be assumed by the SRM 3600 user. However, depending on the X-ray energy used, any intensity uncertainty associated with coupon thickness may be partly mitigated by the measured transmission (determined by absorption and other attenuation effects) both of the standard and of the sample. In any case, this uncertainty, which shows no strong dependence on the plate from which a coupon was cut, or on the position on the plate it was cut from, is small compared to the overall uncertainties in the calibration results, as discussed below.

To determine both measurement repeatability and coupon variability in determining the absolute SAXS intensity over the *Q* range of interest for certification (0.008 < *Q* < 0.25 Å^−1^), the APS USAXS instrument was set up using the APS undulator A (Dejus *et al.*, 1994 ▸) and the beamline Si(111) monochromator optics to select an X-ray energy *E* = 12.0 keV, with an associated X-ray wavelength λ = 1.0332 Å. The calibration of APS undulators and monochromators for X-ray energy and wavelength depends ultimately on measuring the X-ray transmission, as a function of X-ray energy, through standard absorption foils containing known elements. Observed sharp drops in the X-ray transmission are calibrated against the fundamentally determined X-ray absorption energies of the elements present, as determined from NIST traceable look-up tables (Chantler *et al.*, 2005 ▸; Thompson, 2009 ▸). Using this method, the X-ray energy calibration for USAXS measurements has been demonstrated to within a standard uncertainty of ±1.5 eV (Allen *et al.*, 2014 ▸). Measurements were made here with an incident beam size of 0.5 × 0.5 mm.

All 56 coupons were measured at least twice using the APS USAXS instrument. At least one coupon from each plate was measured multiple times (10–20 measurements). The USAXS data were reduced to subtract the empty beam (blank) scattering and the data converted to an absolute intensity scale using the primary method described previously (Long *et al.*, 1991 ▸; Ilavsky *et al.*, 2009 ▸). These data were not desmeared (*i.e.* corrected for slit-smearing effects in the plane perpendicular to the diffraction plane). This is because these initial measurements were focused solely on establishing the experimental uncertainties for repeated USAXS measurements and uncertainties associated with coupon variability. The slit-smeared calibrated SAXS intensity (slit-smeared differential scattering cross section), dΣ′/dΩ, as a function of *Q* is given by
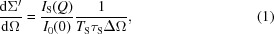
where *I*_0_(0) is the measured intensity without the sample (glassy carbon coupon) present at *Q* = 0, *T*_S_ is the sample transmission, *i.e.* the ratio of the intensity at *Q* = 0 with the sample present to that with no sample present, τ_S_ is the sample thickness and ΔΩ is the solid angle associated with the intensity measurement. The corrected sample (or glassy carbon coupon) scattering intensity, *I*_S_(*Q*), is given by *I*_S_(*Q*) = *I*(*Q*) − *T*_S_ *I*_0_(*Q*), where *I*(*Q*) is the directly measured sample scattering intensity prior to subtraction of the normalized blank intensity, *I*_0_(*Q*). Note that it is the ability of the USAXS photodiode detector to encompass both *I*_0_(0) and *I*(*Q*) within its linear dynamic range for detected X-ray intensity that enables the USAXS instrument to carry out a primary scattering intensity calibration through direct use of equation (1). For USAXS measurements, the normalizing solid angle, ΔΩ, is given by
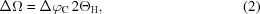
where Δφ_C_ is the angular full width at half-maximum of the analyzing crystal’s rocking curve in the diffraction plane (vertical for the APS USAXS instrument and defined by the crystal diffraction optics used) and 2Θ_H_ is the angle subtended at the sample position by the photodiode detector aperture in the plane orthogonal to the diffraction plane (horizontal for the APS USAXS instrument, and measured directly for each experimental setup).

USAXS data were reduced and calibrated using the *Indra* and *Irena* routines (Ilavsky & Jemian, 2009 ▸) written in *Igor Pro* (Wavemetrics, 2008 ▸). These routines compute estimated standard deviation uncertainties for the intensity *I*(*Q*), measured at each *Q*, but these uncertainties were not used for certification. Instead for any *N* equivalent USAXS measurements, *e.g. N* repeated measurements of the same glassy carbon coupon or measurements, or *N* different glassy carbon coupons selected from the inventory, the results were interpolated and averaged to give the average 〈*I*(*Q*_INT_)〉. Then the mean standard deviation (for one measurement or sample) at each interpolated *Q*_INT_ value, σ_MEAN_(*Q*_INT_), was computed on the basis of the deviation of the *N* actual interpolated *I_i_*(*Q*_INT_) values from the average. Thus
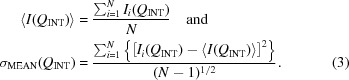
Note, however, that for the standard deviation of the mean result, 〈*I*(*Q*_INT_)〉, itself, σ_MEAN_(*Q*_INT_) must be divided by a further factor, *N* ^1/2^. The data interpolation and point-to-point statistical analyses were conducted using routines developed in MATLAB (The MathWorks Inc., Natick, MA, USA), while overall calibrated intensity comparisons, averaged over the certified *Q* range, were made using the data comparison capabilities of the *Irena* routine package written in *Igor Pro*.

**Figure 3:**
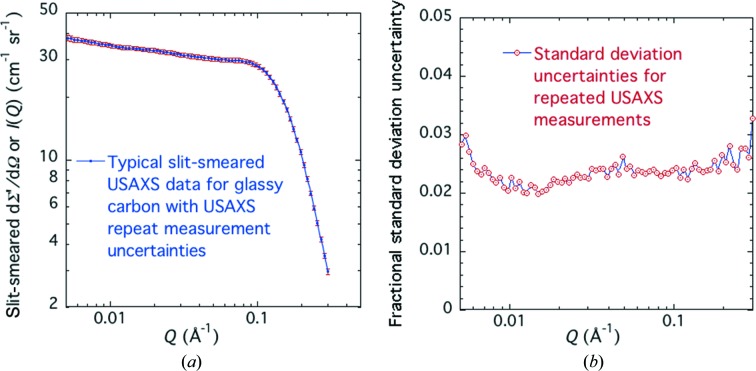
(*a*) Averaged slit-smeared *I*(*Q*) *versus Q* for repeated USAXS measurements. Vertical bars represent standard uncertainties for repeated USAXS measurements. (*b*) Fractional standard uncertainties for repeated USAXS measurements *versus Q*.

Fig. 3 ▸ presents *I*(*Q*) *versus Q* and fractional σ(*Q*)/*I*(*Q*) *versus Q* plots for a typical set of repeated measurements on one glassy carbon coupon. The corresponding plots for the calibrated slit-smeared USAXS intensity data averaged over multiple glassy carbon coupons are similar, except that the fractional uncertainties are slightly larger because these include both uncertainties for repeated measurements and uncertainties for sample variability. However, whether for *N* repeated measurements of the same coupon or for *N* different coupon measurements, the standard uncertainties plotted include uncertainties due to point-to-point variations for different *Q* values. These should not form part of the calibration uncertainty because they are averaged out for data over the certified *Q* range. Thus, for certification purposes, the data management capabilities of the *Irena* USAXS analysis package were used to average datasets over the certified *Q* range, and then to determine the fractional deviations for calibration by testing the normalization of individual datasets with respect to this average. Applying this method to these measurements of the 56 selected glassy carbon coupons, it was found that the fractional standard uncertainty in one glassy carbon coupon intensity calibration was ±2.38% with respect to repeated measurements of the same coupon and ±3.68% with respect to combined measurement repeatability and coupon variation. This assumes that the latter variation is completely random and that the calibration is not correlated with coupon location or plate. In order to test this point a regression analysis was employed.

#### 2.2.1. Regression analysis   

The selection of specimens used to assess different kinds of inter-specimen variation followed an augmented approximation of a central composite experiment design (Agami Reddy, 2011 ▸). This allowed efficient assessment of systematic inter-specimen variation using regression analysis as well as the detection of random inter-specimen variation, if significant. To carry out this analysis, a smoothing spline relating *I*(*Q*) to *Q* was globally fitted to the data from one set of measurements (the first run) across the different glassy carbon plates, omitting the corners. In the absence of spatial variation across the plates, or between plates, such a model would be expected to summarize *I*(*Q*) *versus Q* data well and would look somewhat similar to the curve shown in Fig. 3 ▸(*a*) when viewed on a log scale.

Alternatively, if there were variations in the function relating *I*(*Q*) to *Q*, plate to plate, spatially across the plates or both, then a model with more parameters would be needed to describe the full data set. As one such model that offers more flexibility than the global spline model, which had effectively 28 parameters in *Q*, we chose to fit a full quadratic model in the rows and columns across each plate to the same data from the first run completely locally (*i.e.* by plate, for each value of *Q* individually and for each of two scans over *Q*). In contrast to the global spline model, this model effectively has 3408 parameters (4 plates × 71 parameters in *Q* × 2 scans × 6 parameters for each two-dimensional quadratic model used to capture spatial variation within a plate).

**Figure 4:**
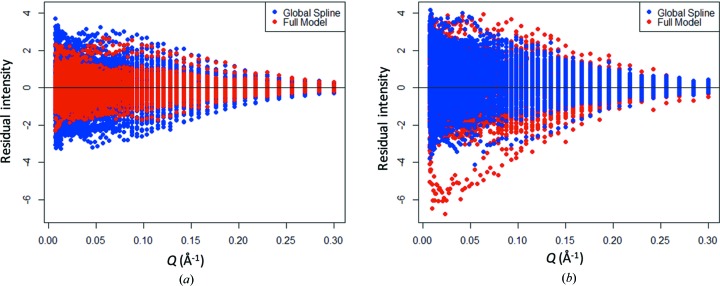
(*a*) Residuals for the fitting or training data from globally fitted smoothing spline (blue) and locally fitted quadratic models (red). (*b*) Residuals for the predictions of new or validation data from globally fitted smoothing spline (blue) and locally fitted quadratic models (red).

Then, to identify potential plate-to-plate or spatial structure in the data, the residuals from these models were compared graphically. If there were significant systematic or random spatial variation between the specimens, the local result would be expected to have smaller residuals than the globally fitted spline model. These two models were then used to predict values of *I*(*Q*) associated with *Q* for the data from the remaining measurement runs over the four plates, and the residuals from these predictions were graphically compared as well. Fig. 4 ▸ shows the residuals from the two models for the fitting data and the validation data (the predicted values).

From Fig. 4 ▸(*a*) one can see that the residuals from the locally fitted quadratic model are smaller, on average, than the residuals from the global spline model. This is an indication of the greater flexibility of the locally fitted full quadratic model. However, from Fig. 4 ▸(*b*) one can see that the two sets of residuals are essentially the same, or if one set of residuals is smaller in magnitude, it is those associated with the global model. The fact that the extra flexibility of the locally fitted full quadratic model does not actually fit the true structure in the data any better than the global spline model does means no extra quadratic like systematic spatial variability is impacting the measurements. Looked at from the other direction, the validation data show that the apparent reduction in the magnitude of the full model residuals in the fitting data is simply caused by the flexible, locally fitted full quadratic model fitting the noise in the data. It is therefore concluded that the specimens from different locations within each plate and across plates are essentially homogeneous, relative to the measurement noise. Note that all but a few of the residuals below a residual intensity of −4 in Fig. 4 ▸(*b*) come from the first measurement pass on coupon 1 on plate A, while the residuals from the second pass on this same coupon were not so different from the fitting data.

One thing to note about Fig. 4 ▸ is that equally weighted fits were used despite the non-constant standard deviation, or heteroscedasticity, visible in these residuals. This is not an issue, however, since all of the fits are local with respect to different values of *Q*. Another potential issue with this analysis is that when these data were collected the measurements were somewhat noisier than expected. The presence of this noise has the potential to limit the ability to detect different types of inter-specimen variation. However, this issue is mitigated by two factors. First, graphical comparisons between specimens using more precise data collected during other phases of the certification analysis did not reveal any evidence of inter-specimen variation. Second, the final uncertainties, which account for USAXS setup uncertainty, also cover the magnitude of the residuals shown in Fig. 4 ▸.

Two additional analyses like this were performed to compare a globally fitted spline model with a quadratic model fitted locally for different values of *Q*, but combining the data from all plates, and with a completely locally fitted mean model, with similar results. These models would help identify other spatial patterns of systematic or random inter-specimen variation, but no such variation was found.

### 2.3. Setup and X-ray energy dependence   

**Figure 5:**
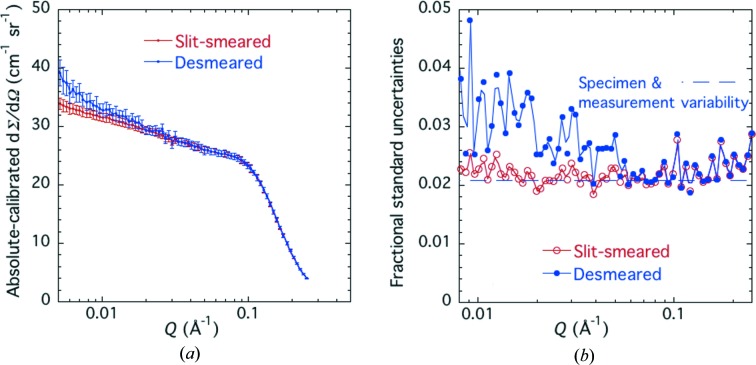
(*a*) Averaged absolute-calibrated dΣ/dΩ *versus Q* for the slit-smeared and desmeared cases for X-ray energy = 11.5 keV. Vertical bars are averaged standard uncertainties. (*b*) Corresponding fractional standard uncertainties *versus Q*.

Having established that no significant correlation exists between measured calibrated intensity and either the plate from which a coupon was cut or its position within that plate, at least to better than the ±3.68% fractional standard uncertainty of the measurements, a smaller number of coupons were selected to determine the uncertainties due to USAXS setup or X-ray energy. To compare data collected using different USAXS setups or X-ray energies, the slit-smeared USAXS data must be desmeared. This was done using the well established slit-desmearing algorithm developed by Lake (1967 ▸), incorporated into the *Irena* data analysis package. To desmear the USAXS data in any group of *N* datasets, the same procedure as described above was employed in order to determine the actual standard uncertainties in each slit-smeared dataset using equation (3). For certification purposes, this is important to ensure the correct criteria for successful desmearing using the Lake routine. To evaluate the *N* desmeared calibrated datasets, these were again averaged and the standard uncertainties evaluated at each interpolated *Q* value, also using equation (3) for the desmeared data. Desmearing corrects for slit-smearing effects by incremental inverse (or reverse) smearing of the data until a slit smearing of the ‘desmeared result’ produces the starting slit-smeared data to within the data residuals. Unfortunately, this process also increases the point-to-point scatter in the desmeared data. These effects are shown in Fig. 5 ▸. However, when the calibration fractional uncertainties are evaluated by comparing any set of individual desmeared datasets with their average dataset over the certified *Q* range, these are found to be the same as for the corresponding set of slit-smeared datasets. Note that it is the desmeared form of dΣ/dΩ that corresponds to the primary definition of the differential scattering cross section presented earlier.

New measurements were made on selected glassy carbon specimens (between four and 16) at four different X-ray energies: 10.0, 11.5, 12.0 and 16.8 keV. Compared to the earlier measurements used in the regression analysis, key stage motions of the USAXS instrument had been replaced. This resulted in a significant reduction in the measurement uncertainties associated with repeated measurements taken with any one USAXS setup at a given X-ray energy. On comparing individual datasets with the average desmeared calibrated USAXS dataset, the standard fractional uncertainties for single USAXS measurements at the given energies and setups, calculated using equation (3), are obtained, as shown in Table 1 ▸.

Averaging the uncertainties for the four energies, using just the measurements of four coupons common to each energy (square root of the average of the four variances), we find an average fractional standard uncertainty of ±2.11% over the certified *Q* range, while a weighted pooled average using the deviations from the respective averages at each energy gives an average fractional standard uncertainty of ±1.96%. Either way these are close to that found for 11.5 keV, indicated in Fig. 5 ▸(*b*) by the horizontal blue dashed line. These uncertainties include measurement repeatability and coupon variation, and they are indeed significantly smaller than those found in the earlier measurements prior to replace­ment of the USAXS stage motion. However, these uncertainties do not include those actually associated with the USAXS X-ray energy and configuration setup, which are more significant.

Several factors create uncertainties in the intensity calibration of all USAXS data obtained with a given setup and USAXS energy. While the X-ray energy, itself, is calibrated to within a few eV and both the analyzing crystal rotation angle, φ, and the analyzing crystal Darwin width, Δφ_C_, are well defined with negligible uncertainty for calibration purposes, the angle, 2Θ_H_, subtended by the detector slit length at the sample requires a physical measurement of the path length from the sample through the analyzer crystals to the photodiode detector. Typically, this is ∼900 mm with an uncertainty of less than 10 mm. This suggests a fractional uncertainty in the solid angle, Δφ_C_2Θ_H_, in equation (2) of ∼1%, which remains small compared to other uncertainties.

More significant uncertainties arise from any finite crystal tilt angles – within each of the collimating and analyzing crystal stages (Fig. 1 ▸) – between their first and second crystals, and also between the respective overall planes of diffraction for the collimating and analyzing stages. Symmetrically cut crystals are used in both the collimating and analyzing crystal monoliths. For each crystal pair, it is important to ensure that the crystal planes of the two crystals are parallel. Obviously, this must be true in the diffraction plane for the Bragg diffraction condition to be satisfied. It has also been shown previously (Ilavsky *et al.*, 2009 ▸) that any nonzero tilt angle of one analyzing crystal relative to the other, in the plane perpendicular to the diffraction plane, causes small changes in the overall incident angle of the beam to the crystal for successive reflections, and hence can cause a reduction in the measured scattering intensity to below the true value when compared to the unscattered incident beam intensity at *Q* = 0. Meanwhile, if the diffraction plane of the analyzing stage is twisted slightly in azimuthal angle from the diffraction plane of the collimating stage, the transmitted intensity at *Q* = 0 can be reduced to below its true value. In this case, because the sample scattering decouples any effect of this azimuthal twisting between the collimating crystals before the sample and the analyzing crystals after the sample, the scattered intensity, itself, is not reduced. Thus, when measured relative to the unscattered incident beam intensity at *Q* = 0, the measured scattered intensity appears to be greater than its true value. It should also be noted that these crystal tilt effects are additive with any scattered beam divergence associated with the finite slit length in the plane orthogonal to the diffraction plane.

The crystal tilts are minimized in the plane orthogonal to the diffraction plane as the crystals are aligned during setup at a given X-ray energy by using removable picomotors to ensure that each successive diffracted beam through the crystals is found in the same vertical plane as the incident beam. Because there are multiple (usually four) crystal reflections within each of the collimating and analyzing crystal pairs, the effect of any nonzero tilt angle gets amplified on successive reflections. By comparing the small Darwin width of the crystal rocking curve with the angular displacement from the Bragg condition caused by a nonzero tilt angle in the orthogonal plane, and also with the estimated accuracy with which the tilt angles can be zeroed (typically, ±0.002°), it can be estimated that the intensity calibration uncertainty associated with nonzero crystal tilts is typically a few percent (Ilavsky *et al.*, 2009 ▸).

**Figure 6:**
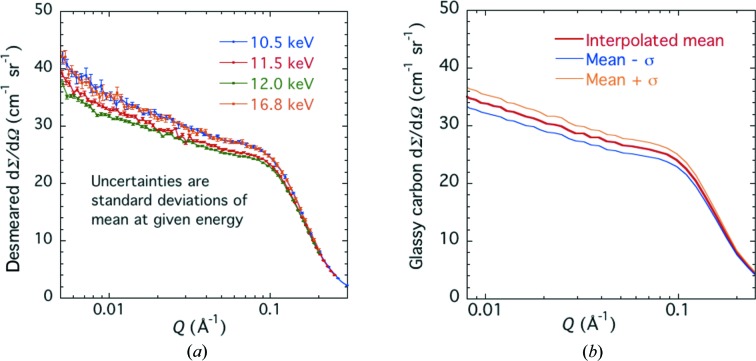
(*a*) Averaged desmeared USAXS intensity *versus Q* for four different X-ray energies (vertical bars are standard uncertainties for averaged data plotted). (*b*) Glassy carbon USAXS intensity calibration curve showing the standard uncertainty band for one measurement of one glassy carbon coupon with one USAXS setup and energy.

Both to assess the calibration uncertainties associated with these effects and to determine if there was any systematic calibration dependence on the X-ray energy, the averaged, desmeared, calibrated USAXS data for the measurements at each X-ray energy were, themselves, averaged. Fig. 6 ▸(*a*) presents the four interpolated and averaged calibration curves, one associated with each X-ray energy (16 datasets averaged at 11.5 and 12.0 keV). The vertical bars represent uncertainties for these averages and include uncertainties due to point-to-point intensity variations with *Q*. No systematic variation with X-ray energy was observed here, and *ad hoc* setups at these and other energies on other occasions indicated a setup variability comparable to that shown here, regardless of the X-ray energy used. The deviations of the average calibrated intensities at each energy from the global average were determined using (once again) the data management capabilities of the *Irena* analysis package, and the standard uncertainty for setup at one X-ray energy was found [using equation (3)] to be ±4.27%, clearly the most significant uncertainty in the calibration. On convoluting this with the standard uncertainty for combined coupon variation and measurement repeatability, we find the overall standard uncertainty for a single measurement for one coupon with one USAXS setup at one energy to be ±4.76%. Fig. 6 ▸(*b*) presents the global average dataset with this overall standard uncertainty bound. However, for reasons discussed below, this does not, as yet, represent the final certified result.

### 2.4. Two-dimensional SAXS comparison   

Uncalibrated pinhole geometry two-dimensional SAXS measurements were carried out on a 16-coupon subset of the glassy carbon coupons, coming from a range of center, edge, corner and intermediate locations on the original plates. Although not absolute-intensity calibrated, data were normalized to the incident beam intensity (at least by counting time) on an arbitrary scale, so that data from different specimens could be directly compared. Two-dimensional SAXS measurements were made for the following purposes: (i) Two-dimensional SAXS data exhibit conventional counting statistics, and smoother data can be obtained over some of the certified *Q* range than obtainable with USAXS directly. (ii) Owing to the smaller statistical uncertainties, a better indication can be obtained for true sample variability. (iii) By normalizing to absolute-intensity-calibrated USAXS data, the maximum *Q* for the intensity calibration can be extended or confirmed, compatible with no significant flat background subtraction being required. (iv) Normalization of pinhole SAXS data to the absolute-intensity-calibrated USAXS data is a prototype for how the SRM 3600 SAXS absolute intensity calibration standard should be utilized.

Pinhole geometry two-dimensional SAXS measurements were made using the Materials Science and Engineering Division Critical-Dimension SAXS (CDSAXS) instrument (Ho *et al.*, 2007 ▸) using its Cu *K*α source (40 kV, 20 mA source, X-ray energy = 8.063 keV, wavelength = 1.5418 Å). This is a custom-designed SAXS instrument that was supplied by Rigaku (Rigaku, Austin, TX, USA), incorporating a Rigaku R-Axis 4++ image plate. Measurements were made with two instrument sample-to-detector configurations: one with an effective *Q* range of 0.03–0.7 Å^−1^ (sample-to-detector distance = 600 mm, counting times = 30 min) and the other with *Q* range 0.02–0.13 Å^−1^ (sample-to-detector distance = 3400 mm, counting times = 3 h). The incident beam size was ∼0.3 × 0.3 mm, and the detector pixel size was 0.1 × 0.1 mm. The two instrument geometry configurations were calibrated (in *Q*) using silver behenate (AgBeh). Normalized empty beam scattering runs were used to subtract out parasitic scattering effects, and the two-dimensional SAXS data were reduced using the *Nika Igor Pro* (Ilavsky, 2012 ▸) analysis package.

The data from the two configurations were circularly averaged and merged together for each coupon measured. Unfortunately, parasitic slit scattering effects precluded the inclusion of SAXS data for *Q* < 0.03 Å^−1^ in any comparison with the USAXS data. However, for higher *Q* values, the SAXS data were easily normalized to the USAXS data profile, and the data counting statistics were sufficient to render the statistical uncertainties in the circularly averaged pinhole SAXS data negligible. Indeed, when the SAXS data for the 16 coupons were interpolated and averaged using the *Irena* analysis package, the standard deviation of the individual SAXS dataset scattered intensities from the average, over the measured *Q* range, was only ±0.89%. This is less than the standard uncertainty of ±1.14% found in the measured coupon thickness. These uncalibrated SAXS intensities are not normalized either to coupon thickness, τ_S_, or to sample transmission, *T*_S_. At this X-ray energy, the variation in *T*_S_ partly cancels out variations in τ_S_; so, this reduced observed variability is not surprising. More generally, this result strongly suggests that the contribution of the sample variability to uncertainties in the USAXS calibration is no more than that attributable to variations in τ_S_.

**Figure 7:**
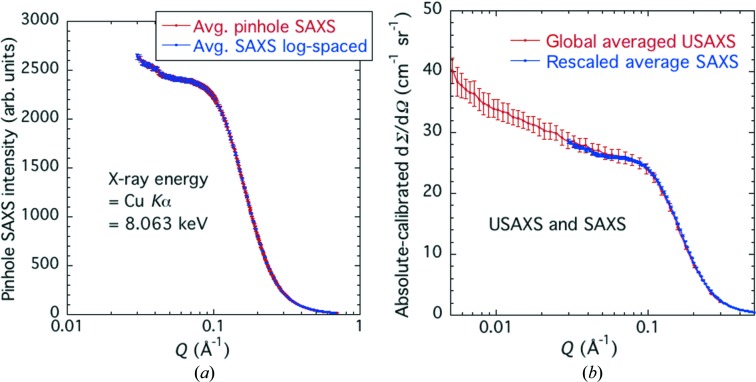
(*a*) Averaged and interpolated pinhole SAXS data for *Q* > 0.03 Å^−1^. Data both linearly binned and log binned in *Q* are shown. (*b*) Uncalibrated pinhole SAXS data rescaled to absolute-intensity-calibrated USAXS data. Vertical bars are standard uncertainties from all sources (USAXS) and from sample variability (SAXS).

Fig. 7 ▸ presents both the averaged pinhole SAXS data for *Q* > 0.03 Å^−1^ and these same data rescaled and matched to the calibrated USAXS data. Apart from the points made above, it is also evident that the maximum *Q* chosen for certification represents a good compromise between maximizing the *Q* range and ensuring sufficient scattered intensity such that further subtraction of flat background scattering is not required for calibrating a typical pinhole SAXS instrument.

### 2.5. Independent validation by SANS   

Although the measurements and uncertainties discussed in previous sections are sufficient to establish the SAXS intensity calibration of SRM 3600, the SAXS intensity calibration must also be validated using an independent measurement. SANS measurements were chosen for validation because the small-angle scattering originates from the same morphology as SAXS, and specifically from the scattering contrast between the glassy carbon ribbons and voids within the microstructure. The scattering contrast factors for either X-rays or neutrons, between glassy carbon and voids, can be determined from the skeletal density and composition of the solid glassy carbon ribbons, look-up tables of the X-ray form factors traceable to NIST (Chantler *et al.*, 2005 ▸; Thompson, 2009 ▸), and neutron scattering lengths (Sears, 1992 ▸; NIST, 2013 ▸). In fact, for rescaling SANS results to SAXS, not even the glassy carbon ribbon density is required if it can be assumed that the composition is the specified 100% pure carbon, as the density cancels out on rescaling SANS to SAXS. Whereas the SAXS intensity calibration of the glassy carbon coupons has been obtained from the previously described primary absolute calibration of the USAXS measurements, the SANS intensity must be calibrated using known calibrated attenuators. In principle, the two-dimensional SANS geometry can be used to determine the scattering probability as a function of *Q*. In practice, most two-dimensional SANS detectors would be damaged, or at least would saturate, if exposed to the non-attenuated incident beam. So, calibrated attenuators must be used to reduce the incident beam intensity.

SANS measurements were carried out on eight glassy carbon coupons: four cut from the centers of the original glassy carbon plates, and four from intermediate regions between the centers and edges. The SANS measurements were carried out using the NIST/NSF NG3 30 m SANS instrument, now relocated to neutron guide NGB (Glinka *et al.*, 1998 ▸) at the NIST Center for Neutron Research (NCNR). A sample thickness of 1.055 mm was assumed for all eight glassy carbon specimens. A neutron wavelength, λ, of 5.05 Å was used, with Δλ/λ = 13.1%. The sample aperture, defined by a Cd mask positioned in front of each specimen, was 6.3 mm in diameter. Three sample–detector measurement configurations were used, with sample-to-detector distances of 2.0 m (with a 25 cm offset of the 64 × 64 cm detector to increase the maximum *Q* measured), 5.0 m and the maximum 13.17 m. In order to approximately match the incident beam collimation conditions with those for the scattering flight path (detector pixel dimension subtended at the sample), one, four and seven neutron guides were used in the incident beam path for sample-to-detector distances of 13.17, 5.0 and 2.0 m, respectively. A 50.8 mm beam-stop was used during all the scattering runs. Data were reduced, calibrated and circularly averaged for each measurement configuration, separately, using the NCNR SANS data reduction package also written in *Igor Pro* (Kline, 2006 ▸). Then, the three one-dimensional datasets associated with each coupon were inter-normalized and merged using the SANS data reduction package to obtain a single one-dimensional SANS dataset covering a *Q* range from 0.0046 to 0.39 Å^−1^. While data for the three SANS instrument configurations were calibrated separately (*i.e.* independently) and the inter-normalization factors were close to one, the one-dimensional dataset corresponding to the largest sample-to-detector configuration (data at smallest *Q* values) was used as the primary file for calibration. Thus, the overall SANS intensity calibration was effectively with respect to the most tightly collimated incident neutron beam (longest incident and scattered collimation distances). This situation most closely resembles the USAXS absolute intensity calibration with X-rays.

**Figure 8:**
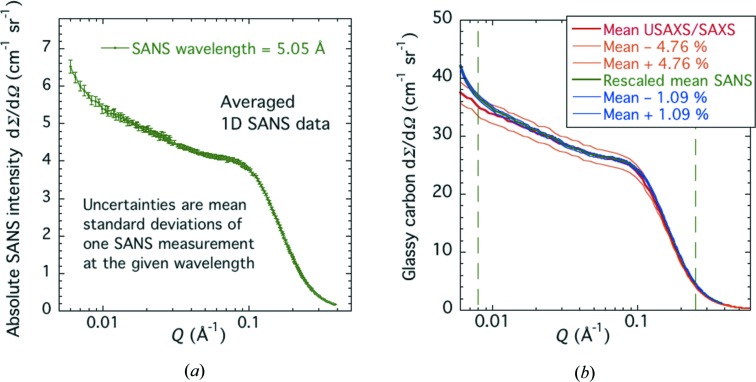
(*a*) Absolute-calibrated mean SANS intensity *versus Q*. Vertical bars are standard deviation uncertainties. (*b*) Comparison of absolute-calibrated SANS intensity for glassy carbon, rescaled for X-rays, and absolute-calibrated USAXS/SAXS intensity *versus Q* from USAXS calibration. Fractional standard deviation uncertainty bands are shown for both datasets. Vertical dashed lines indicate the *Q* range for certification.

The SANS intensity was averaged for all glassy carbon coupons measured, and the mean fractional standard deviation in the intensity within the *Q* range for certification (0.008 < *Q* < 0.25 Å^−1^) was calculated using equation (3). The fractional standard uncertainty for the SANS result, based on a combination of coupon variability and measurement repeatability, was ±1.09%. The averaged SANS intensity data are presented in Fig. 8 ▸(*a*). To compare with USAXS/SAXS data, the SANS data must be rescaled by the ratio of the X-ray and neutron scattering contrast factors for the carbon–void interface within the glassy carbon microstructure. Using the look-up tables for carbon, the X-ray atomic form factor is close to 1.683 × 10^−14^ m, while the neutron scattering length is close to 0.665 × 10^−14^ m. A precise ratio of the square of the look-up values gives the required scattering contrast scaling factor: 6.409. Rescaled SANS data are compared with the USAXS/SAXS data in Fig. 8 ▸(*b*).

Note that the uncertainties presented with the SANS data in Fig. 8 ▸ are those associated solely with statistical sample variability and measurement repeatability, while those for USAXS/SAXS also include uncertainties associated with the USAXS setup at a given X-ray energy. Even so, the rescaled SANS and USAXS/SAXS data clearly agree to within the uncertainty bands indicated throughout virtually all the certified *Q* range, except at the lowest *Q* values where wavelength and geometric smearing effects for SANS are somewhat greater than for USAXS. Not included here is the fractional calibration uncertainty found for SANS instrument alignment and setup. Based on multiple setups and alignments of the NCNR SANS instruments over many years, the estimated fractional standard uncertainty in the SANS intensity calibration due to setup is ±5.0%. This implies a combined fractional standard deviation uncertainty of ±5.12% for one measurement. Clearly, the agreement between the USAXS/SAXS intensities and the SANS intensities is significantly better than this, and we conclude that the SANS intensity calibration validates that for USAXS/SAXS.

While we validate SRM 3600 with SANS intensity measurements, we only certify SRM 3600 for SAXS intensity calibration. While the SANS intensity results were consistent for the particular SANS instrument configuration used, this may not be true for all SANS configurations. The calibrated scattered intensity is dominated by a broad plateau in the scattering as a function of *Q*. At long neutron wavelengths, this scattering plateau can become extremely broad in solid angle, and multiple scattering effects can occur – even for ∼1 mm thick coupons. Furthermore, any hydrogen present, associated with residual polymer inside the glassy carbon morphology, will reduce the neutron scattering contrast factor, while barely affecting the X-ray scattering contrast factor. The same is true if small amounts of moisture ingress into the glassy carbon over time. Either effect could invalidate a SANS intensity calibration. Very recently, Cappelletti (2016 ▸) and co-workers have shown using neutron prompt gamma analysis that the Alfa Aesar Type 2 glassy carbon plate, used as feedstock for SRM 3600, contains virtually no hydrogen, almost certainly as a result of the high pyrolysis temperature used in its manufacture. In view of this point, the possible inclusion of SANS intensity calibration may be considered as part of any future recertification (renewal) of SRM 3600.

## 3. Certification results   

So far, the calibrated intensity data have been given with standard uncertainties applicable to a single measurement on a single glassy carbon coupon. For certification purposes, we need to calculate 95% confidence uncertainties in the calibration curve provided. Meanwhile, uncertainties in the final calibration result can be reduced by appropriate averaging of multiple measurements and setup conditions, but not by averaging of multiple samples (coupons).

### 3.1. Development of certified calibration curve   

We recall that, as part of the certification of SRM 3600, 56 coupons were selected, cut from the centers, edges, corners and intermediate regions of the original glassy carbon plates procured from Alfa Aesar. Following USAXS measurements at 12.0 keV, some repeated, on all 56 coupons and subsequent data reduction, a full regression analysis using global spline, spatial and local models showed that, both within the measurement uncertainties found at the time (fractional standard uncertainty: ±3.68% for one sample measurement) and within the overall uncertainties now established for SRM 3600, there is no significant dependence of the calibrated intensity on either the plate or the location from which the sample was cut.

For further certification, the slit-smeared USAXS data, intensity calibrated using equation (1), were desmeared using the Lake algorithm, which has been well validated for treating USAXS data for a wide range of scattering systems (Ilavsky *et al.*, 2009 ▸). Owing to upgrade and replacement of a key USAXS instrument stage motion, the repeat measurement uncertainties for a given X-ray energy setup were significantly reduced. Averaged USAXS intensity curves were calculated at four different X-ray energies. At two X-ray energies, the averaged USAXS data were for measurements of four coupons. At the other two X-ray energies, the averaged USAXS data were for measurements of 16 coupons. A weighted pooled average (effectively 36 degrees of freedom), using the deviations from the respective averages at each energy, gives a fractional standard uncertainty of ±1.96% for the mean curve. This uncertainty includes both the USAXS repeat measurement uncertainty for a given X-ray energy and USAXS setup, and also sample variability. To distinguish the latter, we note that the two-dimensional SAXS measurements of the relative intensity of 16 glassy carbon coupons showed a fractional standard uncertainty of ±0.89%, *i.e.* less than that in coupon thickness (±1.14%). Taking this as the minimum fractional uncertainty due to sample variability in the USAXS measurements, we find a fractional standard uncertainty in the USAXS intensity calibration, due solely to statistical measurement uncertainties at a given X-ray energy and USAXS setup, of (1.96^2^ − 0.89^2^)^1/2^ = ±1.75%. Although the sample thickness variation is partially cancelled out by the unmeasured sample transmission values, for certification, we conservatively attribute the fractional standard uncertainty due to sample variability to match that of the coupon thickness (55 degrees of freedom).

Uncertainty due to the USAXS setup at a given X-ray energy is clearly the most significant source of uncertainty in the glassy carbon calibration. Based on the USAXS setups at four X-ray energies (three degrees of freedom), the fractional standard uncertainty for one setup is ±4.27%. Thus, the fractional standard uncertainty of one USAXS measurement of one coupon following one USAXS setup at one X-ray energy is ±[(1.14^2^ + 1.75^2^ + 4.27^2^)^1/2^]% = ±4.76%. However, while the ±1.14% sample variability must stand, our calibration result is based on an average of four USAXS setups; so this component uncertainty is reduced to ±(4.27/4^1/2^)% = ±2.14%. Similarly, the uncertainty component for measurements within one setup is based on at least four measurements at each energy; so this component uncertainty in the calibration curve can be reduced to ±(1.75/4^1/2^)% = 0.88%. As a result, the overall fractional standard uncertainty for the calibration curve associated with any one of the glassy carbon coupons is ±[(1.14^2^ + 0.88^2^ + 2.14^2^)^1/2^]% = ±2.58%.

#### 3.1.1. Computation of 95% confidence uncertainties   

For certification, we require a 95% confidence uncertainty, based not only on the components of the standard uncertainty but also on the degrees of freedom used in the sampling. For the various component uncertainties, these are as stated above. Using the procedures set out by Taylor & Kuyatt (1994 ▸) and JCGM (2008 ▸), the effective number of degrees of freedom in this case is 6.290, and the coverage factor required for multiplying the standard uncertainty to obtain the 95% confidence uncertainty *k* = 2.423. Hence, the final overall fractional 95% confidence uncertainty in the SAXS intensity calibration curve is ±6.25%.

### 3.2. Validation with uncertainties based on SANS measurements   

Many of the same points as made above can be applied to the SANS validation measurements. The fractional standard uncertainty of ±1.09% based on measurements of eight coupons (seven degrees of freedom) is more than the ±0.88% for sample variability obtained from uncalibrated but normalized two-dimensional SAXS measurements. This would leave a fractional standard uncertainty SANS measurement repeatability, alone, of ±[(1.09^2^ − 0.88^2^)^1/2^]% = ±0.64% for a single measurement. Given that eight measurements were made, the component fractional standard uncertainty in the averaged data due to measurement repeatability is ±(0.64/8^1/2^)% = ±0.22%. Conservatively, we retain our assumption of overall sample variability having a fractional uncertainty of ±1.14% based on thickness variation. What remains is the SANS instrument and alignment uncertainty. This is estimated to have a fractional standard uncertainty of ±5%, based on many setups and alignments of the NCNR SANS instruments over the past 25 years or so. Since multiple setups were not examined during these measurements, we treat this as a ‘Type B’ uncertainty for a potential systematic error in the SANS setup. We use the full ±5% in our validation results, but with a very large number of degrees of freedom (9999) for computation purposes. Thus, the overall fractional standard uncertainty for the rescaled SANS intensity validation result is ±[(1.14^2^ + 0.22^2^ + 5.00^2^)^1/2^]% = ±5.13%. Using the procedures set out by Taylor & Kuyatt (1994 ▸) and JCGM (2008 ▸), the effective number of degrees of freedom in this case is 7420.87, and the coverage factor for 95% confidence uncertainty *k* = 1.9603. The final overall fractional 95% confidence uncertainty in the rescaled SANS intensity curve is ±10.06%.

Clearly, if ±6.25% uncertainty bands are applied for 95% confidence to the USAXS/SAXS data in Fig. 8 ▸(*b*), and corresponding ±10.06% uncertainty bands are applied to the SANS data, these two datasets are in excellent agreement, very much confirming the validation of the USAXS/SAXS calibration curve using SANS.

### 3.3. Final certified calibration curve with uncertainties   

**Figure 9:**
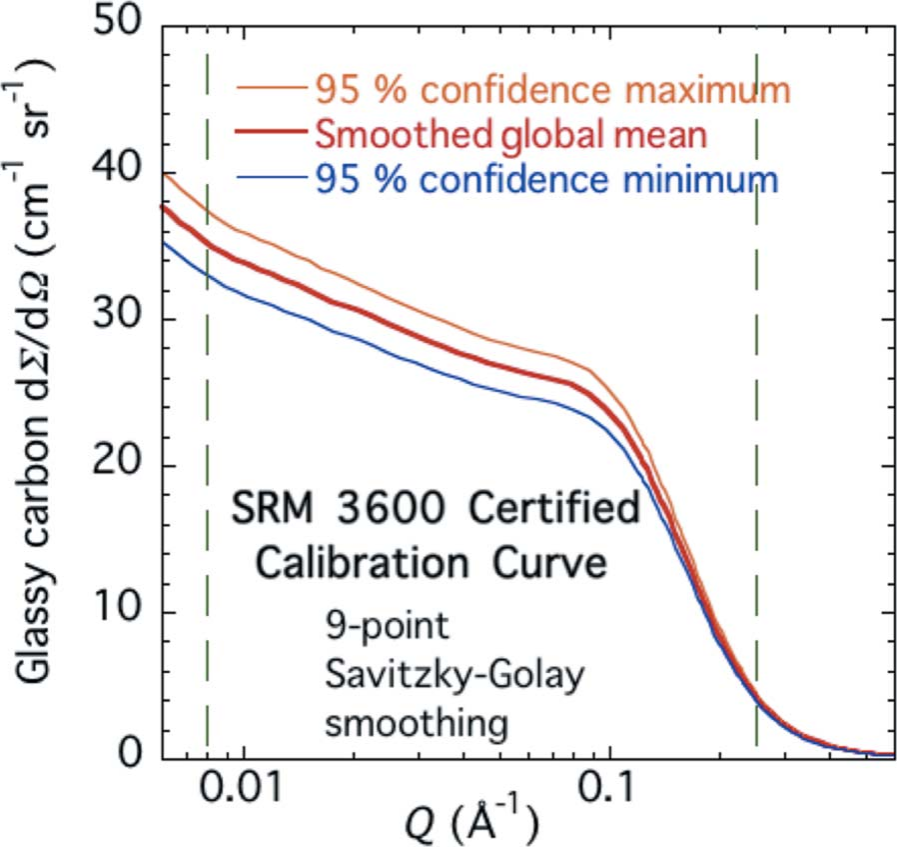
Certified calibrated SAXS intensity *versus Q*. Vertical dashed lines indicate the certified *Q* range.

As a final step to achieve the certified SAXS calibration curve for SRM 3600, nine-point Savitzky–Golay smoothing has been applied to reduce the residual point-to-point scatter in the data (Savitzky & Golay, 1964 ▸). The final certified calibrated intensity data, with 95% confidence uncertainty bands, are presented in Fig. 9 ▸ and are valid for use with all SRM 3600 glassy carbon units.

### 3.4. Use of calibration curve and standard reference material   

The certified calibration curve of dΣ/dΩ *versus Q* is used with an SRM 3600 glassy carbon coupon as follows. Assuming all measurements have been made under the same conditions (including the same incident beam size, collimation and measurement time), the circularly averaged data, 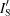
(*Q*) for the sample and 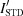
(*Q*) for the standard, are normalized to their respective sampling volumes and are corrected for attenuation in the sample and standard, respectively. The measured 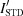
(*Q*) *versus Q* is compared with the certified calibration curve, *I*_STD_(*Q*) = dΣ/dΩ_STD_ *versus Q*, on a log–log scale. If the two curves are parallel over the certified *Q* range, then no further flat background subtraction is required. However, if this is not the case for the data at high *Q*, then a flat background must be subtracted from the experimental data such that the two curves become parallel over the certified *Q* range on a log–log scale. (Some flat background subtraction may also be necessary for the ‘unknown’ sample, but this must be left to the judgment of the user.) On dividing the certified calibration standard curve, *I*_STD_(*Q*) = dΣ/dΩ_STD_ *versus Q*, by the measured curve for the standard, 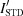
(*Q*) *versus Q*, in the certified *Q* range, *Q* = 0.008–0.25 Å^−1^ (or that part of this range actually measured), the intensity calibration factor, CF, is obtained. The sample data, 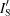
(*Q*), can then be multiplied by the same factor, CF, to convert the ‘unknown’ sample scattering curve to an absolute calibrated scale in differential scattering cross section per unit sample volume, giving a curve for absolute-intensity-calibrated intensity: *I*_S_(*Q*) = dΣ/dΩ_S_ *versus Q*. Note that, even though the calibration standard is measured within the valid certified calibration range of *Q* to determine CF, the calibrated intensity for the ‘unknown’ sample should be valid for all its measured *Q* range.

## 4. Conclusions and availability of NIST SRM 3600   

The certification of NIST Standard Reference Material 3600: Absolute Intensity Calibration Standard for Small-Angle X-ray Scattering has been presented. The Certificate of Analysis with instructions on use and storage of the SRM, the certified intensity calibration data for dΣ/dΩ *versus Q* over the certified *Q* range from 0.008 to 0.25 Å^−1^, and all other data and information files are all freely available at https://www-s.nist.gov/srmors/view_detail.cfm?srm=3600. However, the cer­tification applies only to SRM 3600 glassy carbon units in the inventory. Units of SRM 3600, each of which consists of one SRM 3600 glassy carbon coupon, can be purchased from the NIST Office of Standard Reference Materials, with full details available at the above web site.

## Figures and Tables

**Table 1:** Fractional standard uncertainty in desmeared calibrated USAXS intensity for one measurement of one coupon for a USAXS setup at a specific X-ray energy

X-ray energy (keV)	Number of coupons measured	Standard uncertainty (%)
10.5	4	2.19
11.5	4	2.16
11.5	16	2.09
12.0	4	1.81
12.0	16	1.71
16.8	4	2.24

The same four glassy carbon coupons, each cut from the center of a plate, were measured at all four X-ray energies. At 11.5 and 12.0 keV, 12 additional coupons were measured, cut from the edge, corner and intermediate locations.

## Footnotes

1 Certain commercial instruments, materials or processes are identified in this paper to adequately specify the experimental procedure. Such identification does not imply recommendation or endorsement by the National Institute of Standards and Technology, nor does it imply that the instruments, materials or processes identified are necessarily the best available for the purpose.

2 The use of 55 degrees of freedom here assumes no structure in the thickness measurement results for each coupon (*i.e.* any fluctuations in thickness measurement results from coupon to coupon are assumed to be independent, to be identically distributed and to approximately follow a Gaussian probability distribution).

## References

[bb1] Agami Reddy, T. (2011). *Applied Data Analysis and Modeling for Energy Engineers and Scientists*. New York: Springer Publishing.

[bb2] Allen, A. J., Ilavsky, J., Jemian, P. R. & Braun, A. (2014). *RSC Adv.* **4**, 4676–4690.

[bb3] Bonse, U. & Hart, M. (1965). *Appl. Phys. Lett.* **7**, 238–240.

[bb4] Cappelletti, R. L. (2016). Personal communication.

[bb5] Chantler, C. T., Olsen, K., Dragoset, R. A., Chang, J., Kishore, A. R., Kotochigova, S. A. & Zucker, D. S. (2005). *X-ray Form Factor, Attenuation and Scattering Tables (Version 2.1)*. NIST, Gaithersburg, Maryland, USA, http://physics.nist.gov/ffast.

[bb35] Craievich, A. F. (1976). *MRS Bull.* **11**, 1249–1256.

[bb6] Dejus, R. J., Lai, B., Moog, E. R. & Gluskin, E. (1994). Report ANL/APS/TB-17. Argonne National Laboratory, Argonne, Illinois, USA.

[bb7] Dreiss, C. A., Jack, K. S. & Parker, A. P. (2006). *J. Appl. Cryst.* **39**, 32–38.

[bb8] Fan, L., Degan, M., Bendle, S., Grupido, N. & Ilavsky, J. (2010). *J. Phys. Conf. Ser.* **247**, 012005.

[bb9] Glatter, O. & Kratky, O. (1982). *Small-Angle X-ray Scattering*. New York: Academic Press.

[bb10] Glinka, C. J., Barker, J. G., Hammouda, B., Krueger, S., Moyer, J. J. & Orts, W. J. (1998). *J. Appl. Cryst.* **31**, 430–445.

[bb11] Guinier, A. & Fournet, G. (1955). *Small-Angle Scattering of X-rays.* New York: John Wiley and Sons.

[bb12] Ho, D. L., Wang, C., Lin, E. K., Jones, R. I., Wu, W. L., Seiler, D. G., Diebold, A. C., McDonald, R., Garner, C. M., Herr, D., Khosla, R. P. & Secula, E. M. (2007). *AIP Conf. Proc.* **931**, 382–386.

[bb13] Ilavsky, J. (2012). *J. Appl. Cryst.* **45**, 324–328.

[bb14] Ilavsky, J. & Jemian, P. R. (2009). *J. Appl. Cryst.* **42**, 347–353.

[bb15] Ilavsky, J., Jemian, P. R., Allen, A. J., Zhang, F., Levine, L. E. & Long, G. G. (2009). *J. Appl. Cryst.* **42**, 469–479.

[bb16] Ilavsky, J., Zhang, F., Allen, A. J., Levine, L. E., Jemian, P. R. & Long, G. G. (2013). *Metall. Mater. Trans. A*, **44**, 68–76.

[bb17] Jemian, P. R. & Long, G. G. (1990). *J. Appl. Cryst.* **23**, 430–432.

[bb18] Jenkins, G. M. & Kawamura, K. (1971). *Nature*, **231**, 175–176. 10.1038/231175a0 16062610

[bb19] JCGM (2008). *Evaluation of Measurement Data – Guide to the Expression of Uncertainty in Measurement.* JCGM 100:2008. Joint Committee for Guides in Metrology.

[bb20] Kline, S. R. (2006). *J. Appl. Cryst.* **39**, 895–900.

[bb21] Kostorz, G. (1979). *Small-Angle Scattering and its Applications to Materials Science*, Treatise on Materials Science and Technology, Vol. 15. New York: Academic Press.

[bb22] Lake, J. A. (1967). *Acta Cryst.* **23**, 191–194.

[bb23] Long, G. G., Jemian, P. R., Weertman, J. R., Black, D. R., Burdette, H. E. & Spal, R. (1991). *J. Appl. Cryst.* **24**, 30–37.

[bb24] May, W., Parris, R., Beck, C. II, Fassett, J., Greenberg, R., Guenther, F., Kramer, G., Wise, S., Gills, T., Colbert, J., Gettings, R. & MacDonald, B. (2000). *Definitions of Terms and Modes Used at NIST for Value-Assignment of Reference Materials for Chemical Measurements*, NIST Special Publication 260, p. 136. Washington, DC: US Government Printing Office. http://www.nist.gov/srm/publications.cfm.

[bb25] NIST (2013). *Neutron Scattering Lengths and Cross Sections*, NIST Center for Neutron Research, Gaithersburg, MD, USA, http://www.ncnr.nist.gov/resources/n-lengths/.

[bb26] Orthaber, D., Bergmann, A. & Glatter, O. (2000). *J. Appl. Cryst.* **33**, 218–225.

[bb27] Savitzky, A. & Golay, M. J. E. (1964). *Anal. Chem.* **36**, 1627–1639.

[bb28] Sears, V. F. (1992). *Neutron News*, **3**(3), 26–37.

[bb29] Taylor, B. N. & Kuyatt, C. E. (1994). *Guidelines for Evaluating and Expressing the Uncertainty of NIST Measurement Results*, NIST Technical Note 1297. Washington, DC: US Government Printing Office. http://www.nist.gov/pml/pubs/index.cfm.

[bb30] Thompson, A. (2009). *X-ray Data Booklet.* Berkeley: Lawrence Berkeley National Laboratory.

[bb31] Wavemetrics (2008). *Igor Pro*, http://www.wavemetrics.com.

[bb32] Wignall, G. D. & Bates, F. S. (1987). *J. Appl. Cryst.* **20**, 28–40.

[bb33] Zemb, T., Tache, O., Né, F. & Spalla, O. (2003). *J. Appl. Cryst.* **36**, 800–805.

[bb34] Zhang, F., Ilavsky, J., Long, G. G., Quintana, J. P. G., Allen, A. J. & Jemian, P. R. (2010). *Metall. Mater. Trans. A*, **41**, 1151–1158.

